# Considerations in the selection of patient-reported outcome measures for assessing function related to chronic ankle instability

**DOI:** 10.3389/fbioe.2025.1602283

**Published:** 2025-06-10

**Authors:** Lijiang Luan, Jeremy Witchalls, Phil Newman, Adrian Pranata, Charlotte Ganderton, Oren Tirosh, Doa El-Ansary, Roger David Adams, Gordon Waddington, Jia Han

**Affiliations:** ^1^ Jiangwan Hospital of Hongkou District, Shanghai, China; ^2^ School of Exercise and Health, Shanghai University of Sport, Shanghai, China; ^3^ Research Institute for Sport and Exercise, University of Canberra, Canberra, ACT, Australia; ^4^ School of Health and Biomedical Sciences, STEM College, Royal Melbourne Institute of Technology (RMIT University), Melbourne, VIC, Australia; ^5^ Department of Nursing and Allied Health, Faculty of Health, Arts and Design, Swinburne University of Technology, Melbourne, VIC, Australia; ^6^ Department of Surgery, Melbourne Medical School, University of Melbourne, Melbourne, VIC, Australia; ^7^ Discipline of Physiotherapy, School of Health Sciences, The University of Sydney, Sydney, NSW, Australia; ^8^ Faculty of Health, University of Canberra, Canberra, ACT, Australia; ^9^ College of Rehabilitation Sciences, Shanghai University of Medicine and Health Sciences, Shanghai, China

**Keywords:** chronic ankle instability, patient-reported outcome measures, reliability, validity, responsiveness

## Abstract

Patient-reported outcome measures (PROMs) are essential tools in evaluating chronic ankle instability (CAI), capturing subjective experiences such as “giving way” and instability. However, no standardized guidelines exist for selecting PROMs in CAI, resulting in limited comparability across studies and clinical settings. This paper highlights four key considerations for selecting PROMs in assessing CAI: recalibration in populations including individuals with CAI, identifiability of ankle instability, detectability of CAI characteristics, and cross-cultural adaptability. It emphasizes that CAI-specific PROMs should demonstrate high pertinence, accurately distinguishing CAI from other conditions, and effectively detecting symptom changes over time. Furthermore, widely adopted PROMs may offer greater credibility and applicability. Addressing these considerations is crucial for improving CAI diagnosis, treatment evaluation, and advancing patient-centered care.

## Introduction

Patient-reported outcome measures (PROMs) are standardized tools used to collect patients’ perspectives on their health status, activity limitations, participation difficulties and functional impairments, and they are widely used in health to guide patient-centered care (Houston et al., 2015). In sport rehabilitation, a PROM should closely relate to the specific injury and associated sport-specific impairments, so that the impact of injury on sport participation can be clearly understood, and patient-centered goals be achieved (e.g., return to sport) (Gagnier et al., 2021; Mokkink et al., 2024).

Ankle sprains are the most common sport injury, and lead to a high incidence of chronic ankle instability (CAI) (Hiller et al., 2011; Martin et al., 2021). The use of PROMs in assessing patients with CAI is a common and effective approach for the management of this condition (Houston et al., 2015; Martin et al., 2021). The effectiveness stems from the fact that the primary symptoms of CAI, the sensation of “giving way”, amount of pain and/or feelings of instability, are subjective experiences that are best captured through self-reporting (Houston et al., 2015; Gribble et al., 2014). Currently, a variety of PROMs are employed to evaluate CAI, each offering a different perspective on foot and ankle function, symptoms, and injury severity. However, no guidelines regarding the selection of PROMs for CAI clinical practice and research have been put forward. This is problematic, since the range of instruments employed complicates comparability of findings across studies, diminishing the utility of meta-analyses, hindering the synthesis of evidence, and ultimately affecting clinical decision-making. More importantly, some PROMs may not be specifically tailored to assess CAI symptoms, rendering them less applicable to clinical practice. This situation has the potential to impact on the diagnosis of CAI and the evaluation of treatment effectiveness.

COSMIN is a guideline designed to evaluate the methodological quality of health measurement instruments, and it offers a standardized framework for evaluating the reliability, validity, and responsiveness of PROMs (Mokkink et al., 2024), and plays a valuable role in the assessment (Mokkink et al., 2010; Terwee et al., 2012). Therefore, we believe that PROMs used for CAI should first incorporate the key elements recommended by COSMIN, as these represent the fundamental requirements of a qualified PROM. However, it lacks specificity for certain particular patients. For specialized groups, like those with CAI, a more focused and purpose-driven exploration is needed to ensure that the assessment is tailored and thorough, provides targeted insights for this specific population, and allows for a clearer understanding of its unique challenges. In short, we believe that when evaluating PROMs for CAI patients, merely meeting COSMIN standards is not sufficient; a targeted assessment of the specific symptom characteristics of CAI is also necessary. Accordingly, in this paper we also focus on the limitations of PROMs in assessing CAI, and propose 4 key considerations for use when evaluating current PROMs for the task of assessing CAI.

## The PROMs used for CAI should possess the primary characteristics outlined in COSMIN

The core framework for evaluating measurement instruments, outlined in COSMIN, includes reliability, validity, and responsiveness (Mokkink et al., 2024; Prinsen et al., 2018). PROMs should have relevant supporting data available based on these characteristics highlighted in COSMIN.

Reliability is a critical aspect of evaluating any measurement instrument, as it reflects the consistency and stability of its results across different conditions and time points (Cook and Beckman, 2006; Frost et al., 2007). PROMs with strong reliability provide confidence in their reproducibility (Mokkink et al., 2010). Comprehensive reporting of reliability metrics, such as test-retest reliability, internal consistency, and measurement error, ensures transparency and credibility, which are crucial for the scientific robustness and practical utility of the PROMs (Frost et al., 2007). Without adequate reliability data, the validity of PROM’s outcomes may be compromised, leading to inaccurate interpretations and clinical decisions (Gagnier et al., 2021; Cook and Beckman, 2006; Frost et al., 2007).

Validity refers to the degree to which the measured results reflect the concept intended to be investigated. The more consistent the evaluation result is with the characteristics of the measured object, the more effective the PROM is (Cook and Beckman, 2006; Docherty et al., 2006). Specifically, criterion validity is of particular importance, as it is determined by comparing the PROM’s results with those from an established standard. Also, having good content validity, often assessed by expert review, adds further value to a PROM (Gagnier et al., 2021; Terwee et al., 2012). In particular, the construct (convergent/divergent) validity of the PROM should be noted (Saarinen et al., 2022). Incorporating construct validity data aids researchers in selecting appropriate outcome measures and determines whether a PROM can be effectively used alongside other assessments (Mokkink et al., 2024; Gribble et al., 2014; Sierevelt et al., 2018). Accordingly, a well-developed PROM used in CAI should demonstrate strong construct validity, with adequate data to support its effectiveness (Gagnier et al., 2021; Mokkink et al., 2024). The more robust these data are, the more useful the PROM becomes as a reference for clinicians and evaluators.

Responsiveness refers to a PROM’s ability to detect clinically meaningful changes. For instance, metrics such as the minimum detectable change (MDC) and minimal clinically important difference (MCID) are critical for evaluating the significance of intervention effects (Goldberg et al., 2011; Salas Apaza et al., 2021). If the scoring of the PROM used in the study showed the differences before and after intervention, including statistical differences, but these did not reach the MDC or MCID, then while the results of an intervention study are statistically significant, they may not be clinically significant (Salas Apaza et al., 2021; Bleakley et al., 2021).

Therefore, it is essential that PROMs used in CAI research and clinical practice demonstrate adequate reliability, validity, and responsiveness, supported by sufficient psychometric data to ensure consistent, accurate, and meaningful results across different settings and patient groups.

## The PROMs applied in CAI should address patient-specific attributes

While COSMIN provides a foundational assessment of PROMs, CAI populations require further scrutiny due to their unique clinical profile. PROMs for CAI should address CAI patient-specific attributes to ensure accurate assessment and effective intervention planning. We believe that the following four characteristics are essential: (1) Recalibration in populations including individuals with CAI, (2) Identifiability of ankle instability, (3) Detectability of CAI characteristics, and (4) Cross-cultural adaptability.

(1) A PROM should be tested to obtain its psychometric features in CAI because individuals with CAI have relearning capability and can show variability of feeling (Gagnier et al., 2021; Gribble et al., 2014). For example, functional performance can fluctuate (Gottlieb et al., 2023; Jaber et al., 2018; Ko et al., 2020), as evidenced by improvements in tests like the star excursion balance test (SEBT) over short intervals in individuals with CAI (Ko et al., 2020; Ahern et al., 2021). In addition, one of the main symptoms of CAI (“giving way”) does not occur consistently, and may be experienced sporadically (Hiller et al., 2011). Also, when a significant amount of time has passed since the initial sprain (e.g., at least 12 months prior to study enrollment) (Gribble et al., 2014), the inflammation associated with ankle sprain diminishes, and joint function gradually recovers (Gribble et al., 2016). These factors contribute to the variability of CAI symptoms.

Subjective sensations may also fluctuate, and the feeling of ankle instability is not always present in real-time (Lin et al., 2021; Wikstrom and Song, 2019). At certain stages, if a sense of instability occurs recently, individuals might be consciously wary; however, over time, individuals may forget these sensations if their symptoms are mild and not consistently experienced during sport and daily activities (Hiller et al., 2011; Vuurberg et al., 2018; Terada et al., 2022). In addition, when the feeling of ankle instability occurs, individuals may not pay attention to it during physical activities (Lin et al., 2021; Wikstrom and Song, 2019). Thus there is a possibility of variation in the results of PROMs scored by individuals with CAI at different time points. In brief, if the psychometric properties of a PROM have not been established in a population that includes individuals with CAI, it cannot accurately reflect these specific characteristics of CAI.

Given that the symptoms of CAI can differ significantly from other foot and ankle conditions (Martin et al., 2021; Gribble et al., 2014), and the specific symptoms of CAI, such as the sensation of “giving way” and persistent instability, necessitate a more tailored approach when choosing assessment tools (Gribble et al., 2014), it is necessary for PROMs applied to CAI to include psychometric information specific to the CAI population. For example, in the case of CAI, PROMs should also demonstrate high reliability within CAI populations. Due to the unique nature of CAI symptoms, it is entirely possible that a given PROM may show different levels of test reliability when applied to CAI compared to other foot and ankle conditions. However, many of the available PROMs have not been tested for reliability in the context of CAI, and this undermines their utility. Also, the number of PROMs available complicates the selection process at present, making it difficult to choose a tool that accurately reflects the symptoms and severity of CAI. A primary concern is that many PROMs have not been validated with CAI populations. If validity studies for CAI are limited or lacking, the use of a PROM may be questioned, and the credibility of research findings diminished.

Furthermore, when applied to CAI populations, PROMs that reported MDC and/or MCID for individuals with CAI are better suited for clinical and research applications, which can be seen as a key indicator (Bloom et al., 2023; Seamon et al., 2022), enabling an understanding of the impact of changes (Gagnier et al., 2021; Mokkink et al., 2024). However, not all current PROMs for CAI have sufficient data on these parameters, limiting their utility when assessing treatment outcomes. Without this information, clinicians and researchers may not be able to gauge the true impact of interventions, potentially leading to suboptimal treatment decisions.

In short, the psychometric data for PROMs used in CAI should be obtained from populations that include individuals with CAI. Otherwise, even if the PROM has reported reliability, validity, and responsiveness, it may still be unsuitable for this specific population.

(2) Identifiability refers to a PROM’s ability to distinguish between individuals with CAI and those with other foot and ankle conditions. The importance of this factor arises because CAI patients have distinct characteristics, most notably the chronic feeling of ankle instability and “giving way” (Gribble et al., 2014). Also, unlike other foot and ankle disorders, individuals with CAI only occasionally experience ankle instability over extended periods, without other obvious symptoms or functional deficits. These unique features require assessment tools that can precisely identify and measure these symptoms. Without clear thresholds or criteria that can identify these specific symptoms, a PROM may fail to accurately diagnose CAI; further, accurate identification is essential not only for diagnosis but also for evaluating treatment efficacy, ensuring that interventions are appropriately targeted, and outcomes are correctly interpreted (Martin et al., 2021; Gribble et al., 2014). If a PROM does not identify CAI symptoms or detect CAI phenotypes, this may lead to misdiagnosis or use of ineffective treatment strategies, ultimately compromising patient care.

In brief, a PROM used for CAI should possess robust identifiability. Many existing PROMs lack the necessary specificity to differentiate CAI from other conditions, and this reduces their clinical relevance. PROMs that do not involve the specific impairment observed in CAI are not recommended unless they add new items for identifying ankle instability, given that the sensation of instability and “giving way” is the main characteristic of CAI (Houston et al., 2015; Gottlieb et al., 2023; Jaffri and Saliba, 2021).

(3) The detectability index of a PROM is critical for individuals with CAI, where precise assessment tools are essential for accurate diagnosis and effective intervention planning (Houston et al., 2015; Gagnier et al., 2021; Hale and Hertel, 2005). Metrics such as diagnostic accuracy, including sensitivity and specificity at specific cut-off points, and floor and ceiling effects, play a pivotal role in determining a PROM’s utility (Wright et al., 2014). First, diagnostic accuracy is important, as high sensitivity ensures that individuals with CAI are correctly identified, while high specificity prevents misclassification of those without the condition (Docherty et al., 2006; Eechaute et al., 2007). These metrics directly impact clinical decisions, such as the suitability of rehabilitation programs and the evaluation of treatment outcomes (Donahue et al., 2011; Eechaute et al., 2008).

Additionally, ceiling effects are equally important to mention as potential limitations associated with the use of PROMs, as these can occur when a significant portion of respondents score near the top of the PROM, limiting the PROM’s ability to detect improvements when comparing sporting and general populations (Gagnier et al., 2021). For CAI, floor and ceiling effects help identify whether a PROM can capture the wide variability in symptom severity and functional limitations within this population (Donahue et al., 2011; Gottlieb et al., 2022). Further, CAI-specific PROMs must accommodate the unique characteristics of this population, such as the recurrent and activity-dependent nature of symptoms. Comprehensive reporting of these metrics not only enhances the scientific validity of a PROM but also ensures its relevance and applicability in clinical and research contexts for CAI.

(4) The cross-cultural adaptability of an instrument is a valuable reference for researchers wishing to choose the best PROMs, since it not only represents the acceptability of a PROM, but also indirectly reflects the practicality and applicability of PROMs. The ideal PROM used in clinical practice is efficient, sensitive, and easy to administer, and the more people and countries that use the PROM, the more appropriate the PROM may be for research and clinical practice. Also, based on the acceptance of PROMs, research results from various areas would be more highly comparable.

However, even when psychometric data such as reliability is available, variations may still occur across different language versions or population subgroups. For example, the intraclass correlation coefficient (ICC) for the Cumberland Ankle Instability Tool (CAIT) ranges from 0.826 (Japanese version) to 0.979 (Arabic version); also, the reliability of CAIT differs across healthy individuals (0.979), those with CAI (0.873) and those with lateral ankle sprain (0.968) (Korakakis et al., 2019). These discrepancies highlight two important points: first, the broader the linguistic adaptation of a PROM, the more feasible it becomes to compare results across regions and cultures; and second, the more clearly stratified the psychometric evaluations are across different patient groups, the more precisely the clinical applicability of the PROM can be defined. Without such clarity, the generalizability and diagnostic utility of a PROM may remain uncertain, particularly when applied outside of its originally validated context.

Therefore, a PROM that has been translated into multiple languages, widely adopted across various regions, and subjected to numerous retest studies holds greater value for both clinical research and application (Houston et al., 2015; Martin et al., 2021). The broader the range of its use and the larger the number of studies employing it, particularly under different cultural backgrounds, the higher its credibility. Widespread adoption not only identifies a relatively standardized tool for comparative analysis, but also strengthens the overall reliability of research findings, a feature that is essential for advancing the understanding and treatment of CAI (Martin et al., 2021; Gribble et al., 2016; Vuurberg et al., 2018). Conversely, a PROM that is less widely-used, limited to specific groups or regions, or lacking sufficient retest studies should be approached with caution as it may be less suitable for immediate adoption.

## Future directions and recommendations

Given these challenges, it is essential to carefully consider the selection of PROMs for CAI assessment. A PROM that demonstrates strong reliability and validity, has documented responsiveness, and has been recalibrated in the CAI population with proven identifiability and detectability should be prioritized. If a PROM lacks a solid foundation with respect to these relevant research features, its clinical significance with CAI patients would be compromised, and its use may result in misdiagnosis and inaccurate evaluations of treatment outcomes. Additionally, the cross-cultural adaptability of a PROM can serve as a reference indicator, since widely-used PROMs may promote clinical research and practice, reducing the risk of adopting ineffective treatment strategies, jeopardizing patient care, and potentially exacerbating the condition.

Considering these key points, more comprehensive investigations and systematic reviews are needed to identify which PROMs are the most appropriate for CAI assessment. Future research should aim to evaluate the applicability of existing PROMs with CAI populations, ensuring that the tools used are both accurate and clinically meaningful. By refining the instrument selection process and focusing on PROMs that meet the outlined criteria, clinicians and researchers can improve the accuracy of CAI diagnosis and treatment evaluation, and enhance patient-centered care.

While this review outlines four key considerations for selecting PROMs in the context of CAI—recalibration in CAI populations, identifiability, detectability, and cross-cultural adaptability—we acknowledge that further specification of which PROMs meet or fall short of these criteria would enhance its practical utility. Indeed, the inclusion of comparative data such as sensitivity, specificity, cutoff values, or adoption in clinical guidelines would help translate theoretical recommendations into actionable guidance for researchers and clinicians. However, a key limitation in the current literature is the lack of large-scale, CAI-specific systematic reviews and meta-analyses that comprehensively evaluate these aspects. Similarly, cross-cultural validation data—particularly multi-language studies involving commonly used instruments like the CAIT—remain relatively sparse, and no global harmonization framework currently exists to standardize PROM development across languages and regions.

In light of these gaps, we strongly advocate for future research efforts to focus on building an evidence base that includes population-specific psychometric data, large-cohort evaluations, and cross-culturally harmonized PROM development. Such investigations would not only strengthen PROM selection strategies in CAI, but also advance the broader goal of more accurate and personalized patient-reported assessments.

## References

[B1] Ahern L. Nicholson O. O'Sullivan D. McVeigh J. G. (2021). Effect of functional rehabilitation on performance of the star excursion balance test among recreational athletes with chronic ankle instability: a systematic review. Arch. Rehabil. Res. Clin. Transl. 3, 100133. 10.1016/j.arrct.2021.100133 34589684 PMC8463475

[B2] Bleakley C. M. Matthews M. Smoliga J. M. (2021). Most ankle sprain research is either false or clinically unimportant: a 30-year audit of randomized controlled trials. J. Sport Health Sci. 10, 523–529. 10.1016/j.jshs.2020.11.002 33188966 PMC8500808

[B3] Bloom D. A. Kaplan D. J. Mojica E. Strauss E. J. Gonzalez-Lomas G. Campbell K. A. (2023). The minimal clinically important difference: a review of clinical significance. Am. J. Sports Med. 51 (2), 520–524. 10.1177/03635465211053869 34854345

[B4] Cook D. A. Beckman T. J. (2006). Current concepts in validity and reliability for psychometric instruments: theory and application. Am. J. Med. 119, 166.e7–166.e16. 10.1016/j.amjmed.2005.10.036 16443422

[B5] Docherty C. L. Gansneder B. M. Arnold B. L. Hurwitz S. R. (2006). Development and reliability of the ankle instability instrument. J. Athl. Train. 41, 154–158. 16791299 PMC1472648

[B6] Donahue M. Simon J. Docherty C. L. (2011). Critical review of self-reported functional ankle instability measures. Foot Ankle Int. 32, 1140–1146. 10.3113/fai.2011.1140 22381198

[B7] Eechaute C. Vaes P. Duquet W. (2008). The chronic ankle instability scale: clinimetric properties of a multidimensional, patient-assessed instrument. Phys. Ther. Sport 9, 57–66. 10.1016/j.ptsp.2008.02.001 19083705

[B8] Eechaute C. Vaes P. van Aerschot L. Asman S. Duquet W. (2007). The clinimetric qualities of patient-assessed instruments for measuring chronic ankle instability: a systematic review. BMC Musculoskelet. Disord. 8, 6. 10.1186/1471-2474-8-6 17233912 PMC1797175

[B9] Frost M. H. Reeve B. B. Liepa A. M. Stauffer J. W. Hays R. D. Mayo F. D. A. (2007). What is sufficient evidence for the reliability and validity of patient-reported outcome measures? Value Health 10, S94–S105. 10.1111/j.1524-4733.2007.00272.x 17995479

[B10] Gagnier J. J. Lai J. Mokkink L. B. Terwee C. B. (2021). COSMIN reporting guideline for studies on measurement properties of patient-reported outcome measures. Qual. Life Res. 30 (8), 2197–2218. 10.1007/s11136-021-02822-4 33818733

[B11] Goldberg A. Casby A. Wasielewski M. (2011). Minimum detectable change for single-leg-stance-time in older adults. Gait Posture 33, 737–739. 10.1016/j.gaitpost.2011.02.020 21444208

[B12] Gottlieb U. Hoffman J. R. Springer S. (2023). Dynamic postural control in individuals with and without chronic ankle instability-do the modified star-excursion balance test and jump-landing stabilization have the same control mechanism? Phys. Ther. Sport 60, 104–111. 10.1016/j.ptsp.2023.01.011 36758488

[B13] Gottlieb U. Yona T. Shein Lumbroso D. Hoffman J. R. Springer S. (2022). Reliability and validity of patient-reported outcome measures for ankle instability in Hebrew. Med. Sci. Monit. 28, e937831. 10.12659/msm.937831 36146912 PMC9514050

[B14] Gribble P. A. Bleakley C. M. Caulfield B. M. Docherty C. L. Fourchet F. Fong D. T. (2016). Evidence review for the 2016 International Ankle Consortium consensus statement on the prevalence, impact and long-term consequences of lateral ankle sprains. Br. J. Sports Med. 50, 1496–1505. 10.1136/bjsports-2016-096189 27259753

[B15] Gribble P. A. Delahunt E. Bleakley C. Caulfield B. Docherty C. Fourchet F. (2014). Selection criteria for patients with chronic ankle instability in controlled research: a position statement of the International Ankle Consortium. Br. J. Sports Med. 48 (13), 1014–1018. 10.1136/bjsports-2013-093175 24255768

[B16] Hale S. A. Hertel J. (2005). Reliability and sensitivity of the foot and ankle disability index in subjects with chronic ankle instability. J. Athl. Train. 40, 35–40. 15902322 PMC1088343

[B17] Hiller C. E. Nightingale E. J. Lin C. W. Coughlan G. F. Caulfield B. Delahunt E. (2011). Characteristics of people with recurrent ankle sprains: a systematic review with meta-analysis. Br. J. Sports Med. 45 (8), 660–672. 10.1136/bjsm.2010.077404 21257670

[B18] Houston M. N. Hoch J. M. Hoch M. C. (2015). Patient-reported outcome measures in individuals with chronic ankle instability: a systematic review. J. Athl. Train. 50 (10), 1019–1033. 10.4085/1062-6050-50.9.01 26332028 PMC4641540

[B19] Jaber H. Lohman E. Daher N. Bains G. Nagaraj A. Mayekar P. (2018). Neuromuscular control of ankle and hip during performance of the star excursion balance test in subjects with and without chronic ankle instability. PloS one 13, e0201479. 10.1371/journal.pone.0201479 30102713 PMC6089409

[B20] Jaffri A. H. Saliba S. (2021). Does verbal encouragement change dynamic balance? The effect of verbal encouragement on Star Excursion Balance Test performance in chronic ankle Instability. Braz J. Phys. Ther. 25, 617–622. 10.1016/j.bjpt.2021.04.002 34001425 PMC8536856

[B21] Ko J. Wikstrom E. Li Y. Weber M. Brown C. N. (2020). Performance differences between the modified star excursion balance test and the Y-balance test in individuals with chronic ankle instability. J. Sport Rehabil. 29, 748–753. 10.1123/jsr.2018-0078 31629325

[B22] Korakakis V. Abassi M. Kotsifak A. Manai H. AbuEsba A. (2019). Cross-cultural adaptation and psychometric properties' evaluation of the modern standard Arabic version of Cumberland Ankle Instability Tool (CAIT) in professional athletes. PLoS One 14 (6), e0217987. 10.1371/journal.pone.0217987 31185034 PMC6559661

[B23] Lin C. I. Houtenbos S. Lu Y. H. Mayer F. Wippert P. M. (2021). The epidemiology of chronic ankle instability with perceived ankle instability-a systematic review. J. Foot Ankle Res. 14, 41. 10.1186/s13047-021-00480-w 34049565 PMC8161930

[B24] Martin R. L. Davenport T. E. Fraser J. J. Sawdon-Bea J. Carcia C. R. Carroll L. A. (2021). Ankle stability and movement coordination impairments: lateral ankle ligament sprains revision 2021. J. Orthop. Sports Phys. Ther. 51 (4), CPG1–CPG80. 10.2519/jospt.2021.0302 33789434

[B25] Mokkink L. B. Elsman E. B. M. Terwee C. B. (2024). COSMIN guideline for systematic reviews of patient-reported outcome measures version 2.0. Qual. Life Res. 33, 2929–2939. 10.1007/s11136-024-03761-6 39198348 PMC11541334

[B26] Mokkink L. B. Terwee C. B. Gibbons E. Stratford P. W. Alonso J. Patrick D. L. (2010). Inter-rater agreement and reliability of the COSMIN (COnsensus-based Standards for the selection of health status Measurement Instruments) checklist. BMC Med. Res. Methodol. 10, 82. 10.1186/1471-2288-10-82 20860789 PMC2957386

[B27] Prinsen C. A. C. Mokkink L. B. Bouter L. M. Alonso J. Patrick D. L. de Vet H. C. W. (2018). COSMIN guideline for systematic reviews of patient-reported outcome measures. Qual. Life Res. 27, 1147–1157. 10.1007/s11136-018-1798-3 29435801 PMC5891568

[B28] Saarinen A. J. Uimonen M. M. Suominen E. N. Sandelin H. Repo J. P. (2022). Structural and construct validity of the foot and ankle ability measure (faam) with an emphasis on pain and functionality after foot surgery: a multicenter study. J. Foot Ankle Surg. 61, 872–878. 10.1053/j.jfas.2021.12.011 34980532

[B29] Salas Apaza J. A. Franco J. V. A. Meza N. Madrid E. Loezar C. Garegnani L. (2021). Minimal clinically important difference: the basics. Medwave 21, e8149. 10.5867/medwave.2021.03.8149 35380557

[B30] Seamon B. A. Kautz S. A. Bowden M. G. Velozo C. A. (2022). Revisiting the concept of minimal detectable change for patient-reported outcome measures. Phys. Ther. 102 (8), pzac068. 10.1093/ptj/pzac068 35670017 PMC9361333

[B31] Sierevelt I. N. Zwiers R. Schats W. Haverkamp D. Terwee C. B. Nolte P. A. (2018). Measurement properties of the most commonly used Foot- and Ankle-Specific Questionnaires: the FFI, FAOS and FAAM. A systematic review. Knee Surg. sports Traumatol. Arthrosc. 26, 2059–2073. 10.1007/s00167-017-4748-7 29026933

[B32] Terada M. Kosik K. B. McCann R. S. Drinkard C. Gribble P. A. (2022). Corticospinal activity during a single-leg stance in people with chronic ankle instability. J. Sport Health Sci. 11, 58–66. 10.1016/j.jshs.2020.08.008 32866712 PMC8847849

[B33] Terwee C. B. Mokkink L. B. Knol D. L. Ostelo R. W. Bouter L. M. de Vet H. C. (2012). Rating the methodological quality in systematic reviews of studies on measurement properties: a scoring system for the COSMIN checklist. Qual. Life Res. 21 (4), 651–657. 10.1007/s11136-011-9960-1 21732199 PMC3323819

[B34] Vuurberg G. Hoorntje A. Wink L. M. van der Doelen B. F. W. van den Bekerom M. P. Dekker R. (2018). Diagnosis, treatment and prevention of ankle sprains: update of an evidence-based clinical guideline. Br. J. sports Med. 52, 956. 10.1136/bjsports-2017-098106 29514819

[B35] Wikstrom E. A. Song K. (2019). Generic and psychological patient-reported deficits in those with chronic ankle instability: a cross sectional study. Phys. Ther. Sport 40, 137–142. 10.1016/j.ptsp.2019.09.004 31542637

[B36] Wright C. J. Arnold B. L. Ross S. E. Linens S. W. (2014). Recalibration and validation of the Cumberland Ankle Instability Tool cutoff score for individuals with chronic ankle instability. Arch. Phys. Med. Rehabil. 95, 1853–1859. 10.1016/j.apmr.2014.04.017 24814563

